# Study protocol of the HYPER-LIV01 trial: a multicenter phase II, prospective and randomized study comparing simultaneous portal and hepatic vein embolization to portal vein embolization for hypertrophy of the future liver remnant before major hepatectomy for colo-rectal liver metastases

**DOI:** 10.1186/s12885-020-07065-z

**Published:** 2020-06-19

**Authors:** Emmanuel Deshayes, Lauranne Piron, Antoine Bouvier, Bruno Lapuyade, Emilie Lermite, Laurent Vervueren, Christophe Laurent, Jean-Baptiste Pinaquy, Patrick Chevallier, Anthony Dohan, Agnès Rode, Christian Sengel, Chloé Guillot, François Quenet, Boris Guiu

**Affiliations:** 1 Institut de Recherche en Cancérologie de Montpellier (IRCM), INSERM U1194, University of Montpellier, Institut régional du Cancer de Montpellier (ICM), Montpellier, France; 2 Department of Nuclear Medicine, Institut régional du Cancer de Montpellier (ICM), University of Montpellier, Montpellier, France; 3 Department of Radiology, Saint Eloi University Hospital, 80 avenue Augustin Fliche, F-34295 Montpellier, France; 4 Department of Radiology, Angers University Hospital, Angers, France; 5 Department of Radiology, Bordeaux University Hospital, Bordeaux, France; 6 Department of Liver surgery, Angers University Hospital, Angers, France; 7 Department of Nuclear Medicine, Angers University Hospital, Angers, France; 8 Department of Liver surgery, Bordeaux University Hospital, Bordeaux, France; 9 Department of Nuclear Medicine, Bordeaux University Hospital, Bordeaux, France; 10 Department of Radiology, Nice University Hospital, Nice, France; 11 Department of Radiology, Assistance Publique - Hôpitaux de Paris, Cochin Hospital, Paris, France; 12 Department of Radiology, Croix Rousse Hospital, Hospices Civils de Lyon, Lyon, France; 13 Department of Radiology, Grenoble University Hospital, Grenoble, France; 14 Department of Surgery, Institut régional du Cancer de Montpellier (ICM), University of Montpellier, Montpellier, France

**Keywords:** Liver metastases, Portal vein embolization, Venous deprivation, Major hepatectomy, Colo-rectal cancer

## Abstract

**Background:**

In patients undergoing major liver resection, portal vein embolization (PVE) has been widely used to induce hypertrophy of the non-embolized liver in order to prevent post-hepatectomy liver failure. PVE is a safe and effective procedure, but does not always lead to sufficient hypertrophy of the future liver remnant (FLR). Hepatic vein(s) embolization has been proposed to improve FLR regeneration when insufficient after PVE. The sequential right hepatic vein embolization (HVE) after right PVE demonstrated an incremental effect on the FLR but it implies two different procedures with no time gain as compared to PVE alone.

We have developed the so-called liver venous deprivation (LVD), a combination of PVE and HVE during the same intervention, to optimize the phase of liver preparation before surgery. The main objective of this randomized phase II trial is to compare the percentage of change in FLR volume at 3 weeks after LVD or PVE.

**Methods:**

Patients eligible to this multicenter prospective randomized phase II study are subjects aged from 18 years old suffering from colo-rectal liver metastases considered as resectable and with non-cirrhotic liver parenchyma. The primary objective is the percentage of change in FLR volume at 3 weeks after LVD or PVE using MRI or CT-Scan. Secondary objectives are assessment of tolerance, post-operative morbidity and mortality, post-hepatectomy liver failure, rate of non-respectability due to insufficient FLR or tumor progression, per-operative difficulties, blood loss, R0 resection rate, post-operative liver volume and overall survival. Objectives of translational research studies are evaluation of pre- and post-operative liver function and determination of biomarkers predictive of liver hypertrophy**.** Sixty-four patients will be included (randomization ratio 1:1) to detect a difference of 12% at 21 days in FLR volumes between PVE and LVD.

**Discussion:**

Adding HVE to PVE during the same procedure is an innovative and promising approach that may lead to a rapid and major increase in volume and function of the FLR, thereby increasing the rate of resectable patients and limiting the risk of patient’s drop-out.

**Trial registration:**

This study was registered on *clinicaltrials.gov* on 15th February 2019 (NCT03841305).

## Background

In patients undergoing major liver resection, portal vein embolization (PVE) has been widely used to induce hypertrophy of the non-embolized liver in order to prevent small-for-size and post-hepatectomy liver failure. PVE is a safe and effective procedure, but does not always lead to sufficient hypertrophy of the future liver remnant (FLR) [1]. Therefore, several approaches have been proposed to improve PVE:

i. combined technique with subsequent embolization of ipsilateral hepatic artery, was efficient for FLR hypertrophy, but has been abandoned regarding the increased risk of liver abscess
ii. intrahepatic biliary ablation using ethanol but seemed to increase the risk of damage to the bile ducts of the FLR;
iii. the adjunct of hematopoietic stem cells to PVE, which is still under study.

Recently, the ALPPS (associating liver partition and portal vein ligation for staged hepatectomy) procedure has been developed by surgeons. Although a very high rate of liver hypertrophy has been reported [2], ALPPS was demonstrated to tremendously increase perioperative mortality and morbidity [3]. Another approach to improve FLR regeneration when insufficient after PVE consists in embolizing hepatic vein(s) [4]. Indeed, the sequential right hepatic vein embolization (HVE) after right PVE demonstrated an incremental effect on the FLR, but implies two different procedures with no time gain as compared to PVE alone. To optimize the phase of liver preparation before surgery, we developed the so-called liver venous deprivation (LVD) technique, a combination of PVE and HVE during the same intervention. We reported that LVD was safe and provided fast and important hypertrophy of the FLR at 3 weeks [5]. More recently, we showed that LVD could provide marked and very rapid increase not only in FLR volume but also in FLR function [6, 7] assessed with ^99m^Tc mebrofenin hepatobiliary scintigraphy with SPECT which has been validated as a quantitative method for evaluating liver function [8].

## Methods/design

### Aim of the study

The main objective of this randomized multicenter phase II trial is to compare the percentage of change in FLR volume at 3 weeks after LVD or PVE using MRI or CT-scan. Secondary objectives are listed in Table 1. Translational research objectives are i) evaluation of pre- and post-operative liver function and ii) determination of biomarkers predictive of liver hypertrophy**.**

**Table 1:** Secondary objectives of the HYPER-LIV01 trial

Tolerance	
Post-operative mortality	
Post-operative morbidity	
Post-hepatectomy liver failure	
Rate of non-resectability due to insufficient FRL	
Rate of non-resectability due to tumor progression	
Per-operative difficulties (adhesions, pedicular dissection …)	
Blood loss, operating time, transfusions	
R0 resection rate	
Post-operative liver volume	
Overall survival	

### Sample size and follow-up period

Our hypotheses for sample size calculation are based on a systematic review on PVE before liver resection, involving 1791 patients [9]: the mean increase of the FLR volume was 37.9% at 26 days. In our preliminary study [5] and in a more recent paper by Le Roy et al. [10], a mean increase of 53% of the FLR volume was observed after 3 weeks. Therefore, it is realistic to expect a difference of 12% (or more) between the 2 procedures at 21 days. With a standard deviation of 14% in each arm, a two-sided α = 5% and a power of 90%, according to a Student Test, 30 patients have to be randomized by arm. Taking into account that 5% of the patients could not be evaluable, 32 patients have to be randomized per arm. Finally, planned enrollment will be 64 subjects. The expected duration of the recruitment of all patients is 24 months with a minimal duration of the subject participation of 5 months.

### Selection of study population

#### Study population

Subjects aged from 18 years old suffering from liver metastases considered as resectable could be enrolled in this study if inclusion and exclusion criteria are satisfied.

#### Inclusion criteria

Patients eligible for inclusion in this study have to meet all the following criteria:

- Liver metastases considered as resectable from colo-rectal origin (as validated by a multidisciplinary committee with at least one senior hepatic surgeon)
- Percentage of FLR volume < 30%
- Age ≥ 18 years
- General health status WHO 0 or 1
- Estimated life expectancy > 3 months
- Patients whose biological parameters are:
  - Platelets ≥100,000/mm3,
  - Polynuclear neutrophils ≥1000/mm3,
  - Hemoglobin ≥9 g/dL (even transfused patients can be included)
  - Creatininemia < 1.5 N
  - Bilirubinemia ≤2 N
  - AST and ALT ≤5 N
  - PT > 70%
- Reference liver CT-scan or MRI done during the 30 days preceding PVE or LVD
- Written informed consent
- National health insurance cover

#### Exclusion criteria

Patients eligible for this study must not meet any of the following criteria:

- Cirrhosis
- Presence of clinical ascites
- Ongoing participation or participation within the 21 days prior to inclusion in the study in another therapeutic trial with an experimental drug
- Serious non-stabilized disease, active uncontrolled infection or other serious underlying disorder likely to prevent the patient from receiving the treatment
- Pregnancy (βHCG positive), breast-feeding or the absence of effective contraception for women of child-bearing age
- Contraindication to MRI (in the following cases, a CT-scan must be used instead): Pacemaker or neurosensorial stimulator or implantable defibrillator, cochlear implant, ferromagnetic foreign body
- Allergy or contra-indication to iodine contrast agents
- Treatment with anticoagulants (heparin or AVK) that cannot be interrupted for 48 h
- Treatment with anti-platelets that cannot be interrupted for 5 days for aspirin or clopidogrel
- Legal incapacity (persons in custody or under guardianship)
- Deprived of liberty Subject (by judicial or administrative decision)
- Impossibility to sign the informed consent document or to adhere to the medical follow-up of the trial for geographical, social or psychological reasons

#### Randomization

**Fig. 1:**
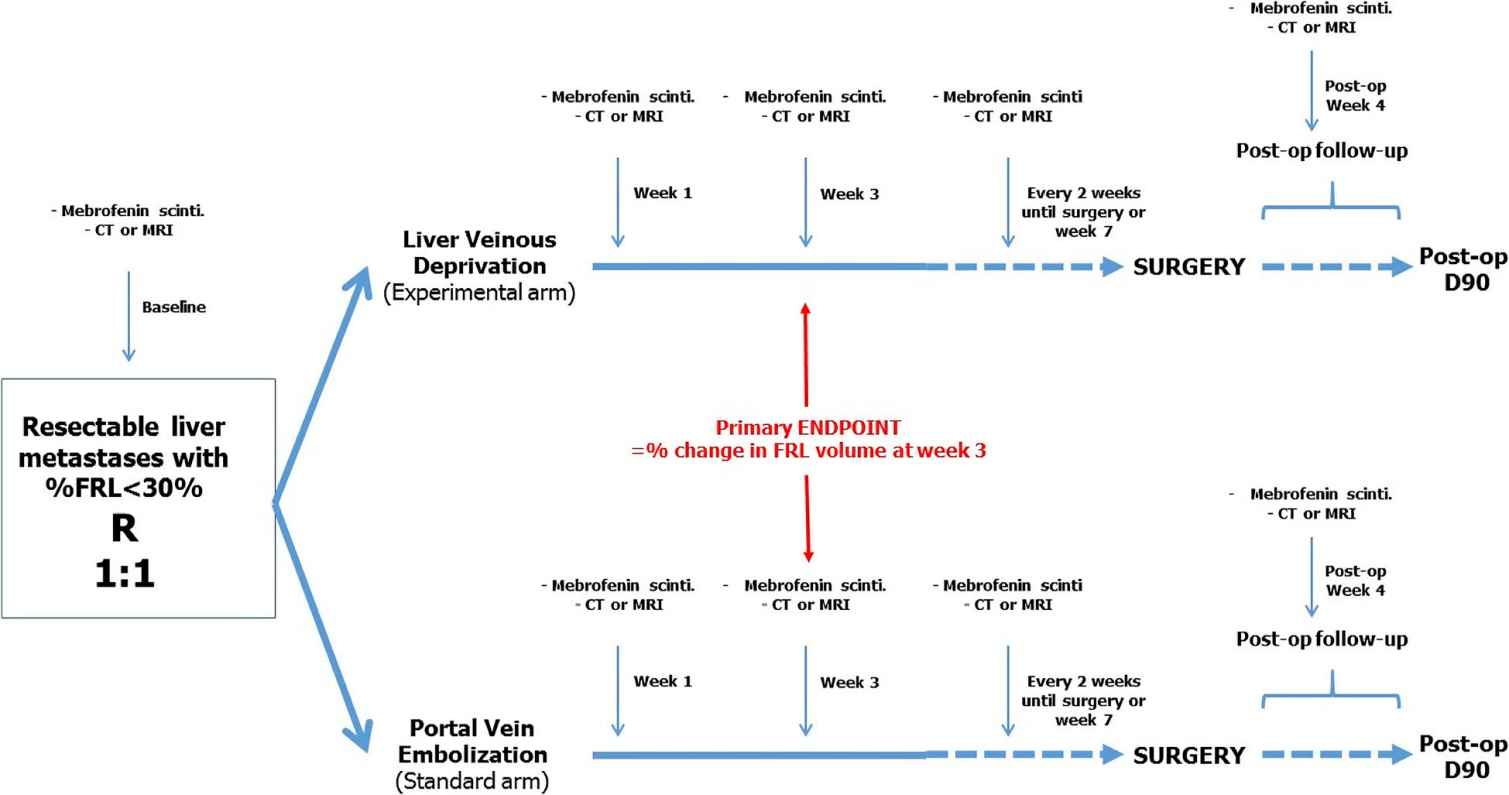
Study design of the HYPER-LIV01 trial

The randomization will be done according to the minimization method (ratio 1:1) and stratified on center and on type of resection scheduled (≤ 4 segments, > 4 segments). The standard arm is Portal Vein Embolization (PVE), the experimental arm is Liver Venous Deprivation (LVD). Figure 1 summarizes the design of the study.

#### Study endpoints

The primary endpoint for the current trial is the percentage of change in future liver remnant (FLR) volume at 3 weeks after liver venous deprivation (LVD) or portal vein embolization (PVE) using MRI/CT-Scan. Secondary endpoints are listed in Table 2. Endpoints for the translational research are:

- Evaluation of pre- and post-operative liver function. This will be evaluated using ^99m^Tc-mebrofenin scintigraphy through SPECT/CT acquisitions by calculating mebrofenin clearance in %/min/m^2^ of whole liver and FLR (described in [8]) at the same time points as CT/MRI (central review).
- To search for biomarkers predictive of liver hypertrophy (EGF, HGF, VEGF, H-EGF, TGF-beta, TNF-alpha, IL-10, IL-6, surviving, FGL-1). Blood samples will be stored by sponsor’s biological resource center (CRB MONTPELLIER). The biological studies on the samples will be managed by a biological committee and funded separately.

**Table 2:** Secondary endpoints of the HYPER-LIV01 trial

Tolerance (toxicities are evaluated according to NCI-CTCAE version 4.03 published 14 June 2010)**.**	
Post-operative mortality defined as any death within 90 days after surgery or within the hospital stay.	
Post-operative morbidity defined as the percentages of grade I/II/II/IV/V complications according to the Clavien-Dindo classification within the 90 days after surgery or within the hospital stay.	
Post-hepatectomy liver failure defined according to the “50–50” criteria *(Balzan, Ann Surg 2005*) or peak bilirubin > 7 mg/dL *(Mullen, J Am Coll Surg 2007).*	
Rate of non-resectability due to insufficient FLR defined as the percentage of patients for whom resection will be not attempted due to insufficient FLR.	
Rate of non-resectability due to tumor progression defined as the percentage of patients for whom resection will not be attempted due to tumor progression.	
Rate of per-operative difficulties defined as the percentage of patients for whom per-operative difficulties are encountered by the surgeon (especially adhesions and challenging pedicular dissection or any other unscheduled surgical difficulties).	
Blood loss, operating time, transfusions. Blood loss (in mL), operating time (in minutes), transfusions (number of packed red blood cells) will be recorded.	
R0 resection rate defined as no microscopic tumor residual.	
Pre- and post-operative liver volumes: This will be evaluated through CT or MRI acquisitions by calculating whole liver, tumor and FRL volumes at week 2, 3 then every 2 weeks until surgery or week #7, and 4 weeks after surgery (central review).	
Overall survival defined as the time from date of randomization to date of death from any cause. Patients alive will be censored at the date of last news.	

### Intervention description

The SPIRIT flow chart is presented in Table 3.

**Table 3:** SPIRIT flow diagram

	Baseline		Liver preparation		After liver preparation			Surgery^**b**^	Post-op follow-up	
			Treatment	During hospitalization	Week 1	Week 3	Every 2 weeks or week 7	During hospitalization	Week 4	Day 90
	D-30 – D0	D-8 – D0	D0	Each day^a^	D0 + 7 days (+/− 1 day)	D0 + 21 days (+/− 1 day)		Each day^c^	Surgery + 28 days (+/− 1 day)	Surgery + 90 days (+/− 1 day)
**Inclusion/exclusion criteria**	X	X								
**Written consent**	X									
**Demographics**	X									
**Medical history**	X									
**Clinical evaluation**	X			X	X	X	X	X	X	X
**ECOG performance status**	X			X	X	X	X	X	X	X
**Prior/concomitant medications**	X			X	X	X	X	X	X	X
**Biological evaluation**		X		X^f^	X	X	X	X^g^	X	X
**Serum pregnancy**		X								
**Biological collection (translational research)**			X^d^	X^d^	X			X^e^		
**Liver biopsy (translational research)**			X					X^h^		
**99 m-Tc mebrofenin scintigraphy (translational research)**	X				X	X	X		X	
**Spiral CT/MRI of abdomen**	X				X	X	X		X	X
**Randomization**		X								
**Liver venous deprivation/portal vein embolization**			X							
**Adverse events/Serious adverse events**				X	X	X	X	X	X	X

^a^ Before and after liver preparation

^b^ Surgery to be performed ≤8 days after the last 99 m-Tc mebrofenin scintigraphy and CT-scan/MRI (except is surgery is performed after week 8)

^c^ Before and after surgery

^d^ The samples of the translational study are to be done the day of the treatment then to D1, D2 and D3 after the treatment. D2 and D3 are optional as soon as the patient is discharged

^e^ The samples of the translational study are to be done the day of surgery

^f^ Biological evaluation are to be done 6 h after treatment

^g^ Biological evaluation are to be done the day before surgery, 6 h and 12 h after surgery, then daily during hospitalization

^h^ Biopsy of the deportalized lobe and FLR are to be done the day of surgery

#### Standard arm (PVE group)

The portal system will be accessed using a micropuncture set either through the left or through the right portal vessels. 2D and/or 3D portography will be performed by inserting a 4F or 5F catheter in the main portal trunk. Portal pressure will be measured. Then portal vessels supplying the future resected liver will be embolized using a mixture of cyanoacrylate and lipiodol (ratio 3–6/1 depending on operator’s preference). If segment IV is scheduled to be resected, PVE of portal vein branches of segment IV is allowed.

#### Experimental arm (LVD group)

If right hemihepatectomy is scheduled: Right hepatic vein as well as accessory right hepatic vein(s) (when present) are accessed using a micropuncture set. After opacification, a 0.018″ microguidewire is left in place in each hepatic vein.

If right hemihepatectomy and resection of segment IV (+/− other segments) is scheduled: Middle & right hepatic veins as well as accessory right hepatic vein(s) (when present) are accessed using a micropuncture set. After opacification, a 0.018″ microguidewire is left in place in each hepatic vein.

Then, the portal system will be accessed using a micropuncture set either through the left or through the right portal vessels. 2D and/or 3D portography will performed by inserting a 4F or 5F catheter in the main portal trunk. Portal pressure will be measured. Then portal vessels supplying the future resected liver will be embolized using a mixture of cyanoacrylate and lipiodol (ratio 3–6/1 depending on operator’s preference). If segment IV is scheduled to be resected, PVE of portal vein branches of segment IV is allowed.

After PVE is completed, microguidewire(s) left in hepatic veins are used to introduce a Neff set. Through the Neff set, a 0.035″ guidewire is inserted to introduce a 7F Destination (Terumo, Japan) sheath in order to deploy an Amplatzer Vascular Plug II (100% oversizing: 14-22 mm) 10-15 mm before the origin of the hepatic vein to keep place for further surgical ligature. After plug deployment, opacification is performed through the sheaths to check for plug occlusion and potential veno-venous collaterals. As soon as the plug is occluded, embolization of distal venous branches is conducted using a mixture of cyanoacrylate (Purefill, Peters Surgical) and lipiodol (ratio 2–3/1). At last, tract embolization is performed using the same mixture. Tract embolization of portal vein access is performed using the mixture used for PVE.

#### Post-procedural prescriptions (both arms)

Pain medication is administered following the recommendations of each center. Morphine administration is allowed.

Day 0: IV multivitamin supplementation.

Day 1: Hydrosol® multivitamin drinkable solution (25 drops morning and evening). Per-os phosphorus supplementation (except if phosphoremia or calcemia > ULN) to maintain phospheremia within the limits of normal.

Day 2: Hydrosol® multivitamin drinkable solution (25 drops in the morning).

## Discussion

We developed an innovative trans-hepatic technique (called LVD) for both PVE and HVE, easy to practice by interventional radiologists. Hepatic vein(s) are accessed under US guidance using micropuncture sets and embolized using Amplatzer vascular plug(s) and cyanoacrylate for distal branches and veno-venous collaterals. In two preliminary studies [6, 7] we showed that LVD is safe (no migration of embolic material was observed) and provided a strong increase in both FLR volume and function at 3 weeks (respectively 52.6% (range, 1–175.6%) and 68.2% (range, 25.4–121.4%)). In a retrospective analysis, we also showed similar mortality/morbidity rates during and after surgery compared to PVE [11]. A paper from another team [12] studying LVD in association with biliary drainage in 6 patients with Klatskin tumors also showed no adverse events and a FLR hypertrophy of 67% (range 29–123) 3 weeks after the procedure. Given the high morbidity and mortality rate following ALPPS [3], LVD could be an attractive alternative technique to increase FLR volume in a short period of time and has the potential to replace PVE as a standard of care. This project also includes functional evaluations of both the whole liver and FLR using 99 m-Tc mebrofenin scintigraphy. This will bring additional useful data given the great potential of liver function to become a more accurate predictor of post-operative liver dysfunction than liver volume.

In conclusion, we believe that LVD is a promising method to improve liver preparation before major hepatectomy, thereby increasing the number of patients undergoing curative surgery and preventing drop-out due to tumor progression. This prospective, multicenter and randomized phase II trial is mandatory to confirm our preliminary results. Serial evaluations of liver function, based on 99 m-Tc mebrofenin scintigraphies, will be helpful to define the optimal time for resection.

## Data Availability

Not applicable.

## Abbreviations

ALPPS: Associating Liver Partition and Portal vein ligation for Staged hepatectomy
ALT: Alanine aminotransferase
AST: Aspartate aminotransferase
FLR: Future Liver Remnant
HVE: Hepatic Vein Embolization
LVD: Liver Venous Deprivation
PHLF: Post-Hepatectomy Liver Failure
PT: Prothrombin time
PVE: Portal Vein Embolization
SPECT/CT: Single Photon Emission Computed Tomography with Computed Tomography
